# Hip Fractures and Hypogonadism in Young Males: A Missed Opportunity for Secondary Prevention?

**DOI:** 10.7759/cureus.90445

**Published:** 2025-08-18

**Authors:** Chan Khin, Olive Kyaw, Shri Thangaraj, Oriade Akinsolu, Amr Elfiky, Prashanth D'Sa, Gareth Chan, Benedict Rogers

**Affiliations:** 1 Trauma and Orthopaedics, University Hospital Sussex National Health Service (NHS) Foundation Trust, Brighton, GBR; 2 Emergency Medicine, South Warwick National Health Service (NHS) Foundation Trust, South Warwick, GBR; 3 Trauma and Orthopaedics, Royal Surrey County Hospital, Brighton and Sussex Medical School, Brighton, GBR

**Keywords:** femoral neck fracture, fragility fracture, hip fracture, men, osteoporosis, testosterone

## Abstract

Introduction: Low testosterone is a known risk factor for osteoporosis and fragility fractures in men. Unlike in women, osteoporosis in men often presents later in life. Fragility hip fractures in males under 60 years are uncommon and may represent an opportunity to diagnose and treat an underlying metabolic bone pathology. This study aims to evaluate whether low-energy hip fractures in young men are associated with low serum testosterone.

Materials and methods: We performed a retrospective review of a prospectively maintained database, identifying male patients aged <60 years who sustained hip fractures from low-energy trauma between January 2022 and December 2024. Fractures up to 5 cm distal to the lesser trochanter were included. Demographic, comorbidity, and laboratory data were collected; serum testosterone <198 pmol/L was defined as low.

Results: Sixty-one males (mean age 50.6 ± 7.2 years) sustained hip fractures in this study period; 23 (38%) had serum testosterone measured. Of those, 15 (65%) were found to have low testosterone; only one had a known endocrine disorder. Among the total cohort, 42 (69%) had multiple comorbidities, 24 (39%) were on polypharmacy (≥5 medications), 25 (41%) were current smokers, 17 (28%) reported excessive alcohol use, and 13 (21%) had a history of substance misuse.

Conclusions: A substantial proportion of young men with fragility hip fractures had unrecognized low testosterone levels. These findings indicate that hormonal abnormalities may be under-recognized in this population, and further studies are warranted to determine whether targeted hormonal screening could help reduce future fragility fracture risk.

## Introduction

Fragility fractures represent a significant public health concern, particularly in light of the growing proportion of the aging population [1]. These fractures, defined as resulting from low-energy trauma, such as a fall from standing height, are common among older individuals. Their occurrence in younger individuals, especially males, is relatively rare. Fragility hip fractures in men under the age of 60 are uncommon and may suggest an underlying pathological bone condition, likely metabolic [2-4]. Pathological conditions can include benign or malignant processes that compromise bone integrity. Male patients who sustain fragility fractures exhibit nearly twice the mortality rate of their female counterparts [5]. This increased mortality may be due, in part, to the delayed diagnosis of osteoporosis in men, which is often only recognized in the presence of comorbidities, thereby complicating treatment. Despite the low incidence of fragility fractures in younger men, this population warrants closer scrutiny due to the associated elevated mortality risk [6].

Delayed recognition of osteoporosis in men, particularly younger males, often results in fractures occurring in the context of untreated or inadequately managed metabolic bone disease. This highlights the importance of early diagnosis and the implementation of preventative strategies to reduce the burden of fragility fractures in this group. One well-established risk factor for osteoporosis in men is testosterone deficiency [7-11]. Testosterone plays a critical role in maintaining bone mass through its stimulation of osteoblast activity. Low testosterone levels can result in decreased bone density, thereby increasing the risk of osteoporotic fractures. A substantial body of literature has established a strong association between testosterone deficiency and hip fractures in older men, identifying it as a key contributor to osteoporosis and fracture risk in this population [7-11]. However, there remains a significant gap in the literature regarding the role of testosterone in younger men, those aged under 60 years, who sustain fragility fractures. This is especially concerning given that young men typically possess higher peak bone mineral density; a fragility fracture in this demographic may reflect a worrying underlying but potentially reversible pathology.

Monitoring testosterone levels in young male patients presenting with fragility fractures may uncover a modifiable risk factor, offering a therapeutic opportunity to reduce both morbidity and mortality [12,13]. MacLean et al. [12] demonstrated that various pharmacological agents used in testosterone replacement therapy can effectively reduce fracture risk and improve bone health in men with documented testosterone deficiency. Therefore, identifying and managing testosterone deficiency in younger men with fragility fractures could have a substantial positive impact on long-term outcomes, including fracture prevention. Routine assessment of testosterone in this group may provide an opportunity to improve clinical outcomes and reduce fracture recurrence.

While low testosterone has been linked to hip fractures in older men, evidence in younger men remains limited. This study is exploratory in nature and aims to address this knowledge gap by investigating the association in males under 60 years of age. Secondary analyses examine the prevalence of lifestyle factors and comorbidities in this population, providing additional context for potential risk factors.

## Materials and methods

Study design

A retrospective analysis of a prospectively collected database of all fragility fractures in males aged 60 years or younger at the time of injury was performed. All sequential patients presenting at a high-volume regional fragility hip fracture unit between January 2022 and December 2024 were eligible for inclusion.

Inclusion and Exclusion

Patients had to have sustained a hip fracture following low-energy trauma, defined as a fall from standing height or less. For this study, a hip fracture was defined as a fracture of the proximal femur extending from the femoral head to a point 5 cm distal to the lesser trochanter, thereby including both intracapsular and extracapsular fracture types [14].

Patients were excluded if the fracture was sustained following high-energy trauma, was classed as major trauma (injury severity score of >15), or had two or more concurrent fractures. High-energy trauma was defined as injury caused by significant kinetic force, such as that sustained in motor vehicle collisions or falls from substantial height.

Study methodology

For eligible patients, medical records were reviewed to extract demographic data and relevant laboratory results. Specifically, serum total testosterone levels obtained at the time of admission were assessed. Serum total testosterone concentrations were measured using an electrochemiluminescence immunoassay on a Roche platform in the hospital laboratory. Free testosterone was calculated from total testosterone, sex hormone binding globulin (SHBG), and albumin concentrations using the Vermeulen equation [15,16]. Testosterone deficiency was defined as a free testosterone level below 198 pmol/L, based on institutional laboratory reference ranges.

Radiological records were reviewed to confirm the fracture was up to 5 cm distal to the lesser trochanter. In cases where low testosterone levels were identified, clinical records were further examined to determine the presence of any pre-existing endocrine disorder that could account for the hormonal abnormality.

Statistical Analysis

Given the small sample size, formal inferential statistical testing was limited. Descriptive statistics were used to summarize patient demographics, comorbidities, and hormone levels. Where appropriate, measures of central tendency (mean, median) and dispersion (standard deviation, range) are presented. Exploratory group comparisons were performed, but these results should be interpreted cautiously due to the limited power. Analyses were conducted using Python version 3.10 statistical libraries (Python Software Foundation, Delaware, United States).

Ethical Approval

The UK Medical Research Council and NHS Health Research Authority decision-making tool was used to ascertain whether this project constituted a research project requiring ethical approval [17]. It was confirmed that ethical approval was not required. This study, therefore, constituted a service evaluation project, and local governance approval was instead obtained.

## Results

Patients' demographics

A total of 61 male patients under the age of 60 sustained femoral neck fractures during the study period. The mean age of the cohort was 50.6 years (range 32-60; SD ±7.18). Of these, 42 patients (69%) had multiple comorbidities. Twenty-four (39%) were prescribed polypharmacy, defined as the regular use of five or more medications. Additionally, 25 patients (41%) were current smokers, 17 (28%) reported excessive alcohol consumption, and 13 (21%) had a history of substance misuse.

Testosterone levels

Of the 61 patients, 23 (38%) underwent serum testosterone level testing upon admission. Of those, 15 patients (65%) were found to have total testosterone levels below 198 pmol/L, consistent with biochemical testosterone deficiency.

Among the 15 patients with low testosterone levels, only one patient had a known pre-existing diagnosis of endocrine deficiency (not hypogonadism). Two additional patients had a documented history of chronic corticosteroid use, one for a diagnosed medical condition and the other for non-prescribed anabolic purposes.

SHBG concentrations were generally within or slightly above the reference range (19-81 nmol/L), whereas serum testosterone levels were markedly reduced in most cases. Total testosterone concentrations were consistently below the normal range of 6.68-25.7 nmol/L. Detailed individual values are shown in Table 1-2.

**Table 1:** Age, comorbidities, and lifestyle of patients with low testosterone IVDU: intravenous drug user

Case number	Age	Comorbidities	Smoking	Alcohol excess	Substance abuse/IVDU
1	55	Asthma, hepatitis B, and splenectomy	Yes	-	Yes
2	52	Type 2 diabetes mellitus, pancreatitis, epilepsy, and general anxiety disorder	Yes	-	-
3	44	Type 1 diabetes mellitus and depression	Yes	-	-
4	55	Ankylosing spondylitis	Yes	-	Yes
5	51	Alcoholic liver disease and hepatic encephalopathy	Yes	Yes	-
6	50	Fit and well	Yes	-	-
7	47	Hepatitis C, alcoholic cirrhosis, deep vein thrombosis, and anxiety	Yes	Yes	Yes
8	54	Lower limb paraplegia following spinal abscess, chronic myeloid leukaemia, previous opioid addiction, schizoaffective disorder, and asthma	-	-	-
9	35	Fit and well	-	-	-
10	57	Chronic obstructive pulmonary disorder, pulmonary emphysema, type 2 diabetes mellitus, common iliac artery aneurysm, sigmoid colon perforation, vitamin D and B12 deficiency, and paranoid schizophrenia	-	Yes	Yes
11	59	Hypotension and osteoporosis	-	Yes	-
12	45	Oesophagogastrectomy for oesophageal carcinoma, asthma, and depression	Yes	Yes	Yes
13	59	Asthma/chronic obstructive pulmonary disease, gastro-oesophageal reflux disease, and depression	-	-	-
14	60	Moderate learning disability, asthma, hypertension, type 2 diabetes mellitus, and depression	Yes	-	-
15	45	Mild learning disability, hypertension, and hypothyroidism	-	-	-

**Table 2:** Testosterone profile of included cases * New reference range SHBG: sex hormone binding globulin

Case number	SHBG (normal range: 19.3-76.4 nmol/L)	Serum testosterone level (normal range: 163-473 nmol/L)	Total testosterone level (normal range: 6.68-25.7 nmol/L)
1	64	62	4.9
2	41	74	4.3
3	34	168 (*198-619)	8.2 (*8.64-29)
4	27	47	2.1
5	78	16	1.5
6	33	151	7.7 (*8.64-29)
7	80	17.1	1.7
8	28	19	0.9
9	19	122	4.8
10	57	1	0.1
11	53	50	0.3
12	33	26	1.3
13	81	144	13.5
14	26	21	0.9
15	28	119	5.5

**Figure 1:**
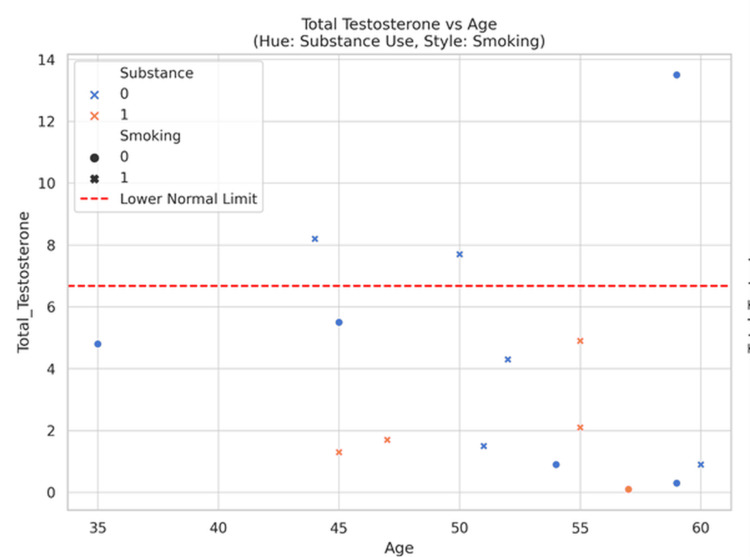
Scatter plot showing total testosterone versus age

Total testosterone concentrations were predominantly below the lower limit of normal across the observed age range, with no strong age-related trend apparent. Substance use and smoking status did not appear to markedly affect this distribution (Figure 1).

**Figure 2:**
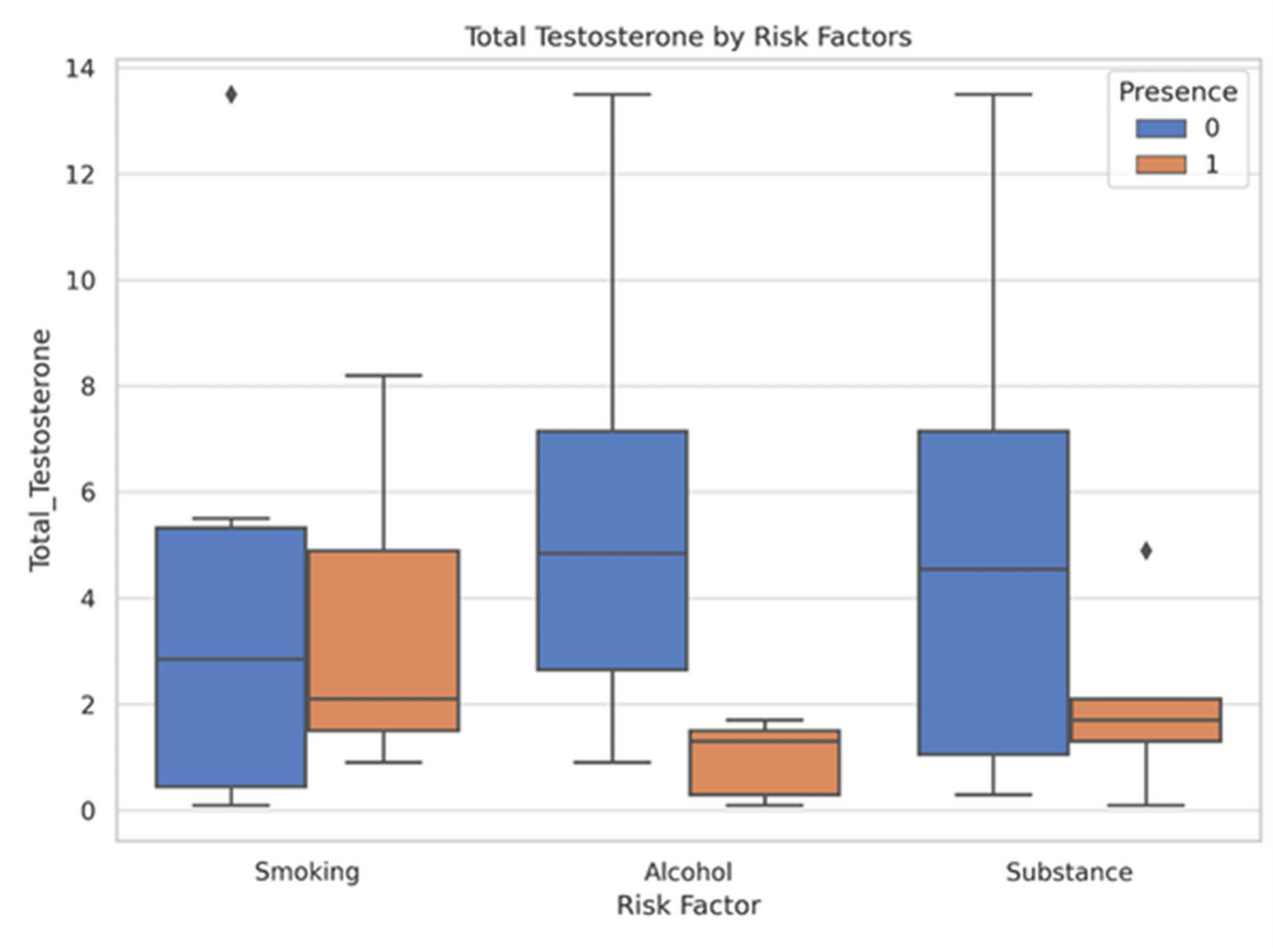
Grouped boxplot showing testosterone levels by substance abuse

Patients reporting smoking, alcohol use, or other substance use exhibited lower median total testosterone levels compared to those without these exposures. This difference was especially pronounced for alcohol and substance use (Figure 2).

**Figure 3:**
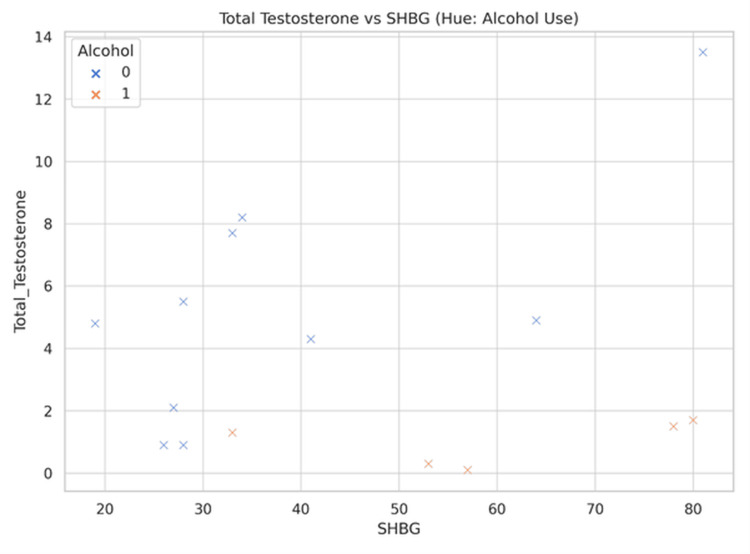
Scatter plot showing SHBG versus total testosterone in relation to alcohol excess SHBG: sex hormone binding globulin

A trend toward lower total testosterone and SHBG concentrations was observed among patients with alcohol excess relative to non-users, although considerable variability existed (Figure 3).

Given the small sample size, formal statistical comparisons between groups were not performed. Results are presented using descriptive statistics. Patients with alcohol use showed lower mean total testosterone levels compared to non-users. Although a statistical test suggested a difference (p=0.006), these results should be interpreted cautiously given the very small sample size (Table 3).

**Table 3:** T-test results for lifestyle factors

Factor	p-value	t-statistic
Smoking	0.81	-0.25
Alcohol	0.006	-3.34
Substance	0.102	-1.8

No meaningful correlation was observed between SHBG and total testosterone (r=0.14, p=0.61) or age and total testosterone (r=-0.13, p=0.65), although this analysis is limited by sample size (Table 4). These findings suggest that excessive alcohol consumption may be a clinically relevant factor associated with suppressed testosterone levels in this population. In contrast, smoking, substance use, and age did not show a clear statistical relationship in this small subgroup.

**Table 4:** Pearson correlations for comparisons SHBG: sex hormone binding globulin

Comparison	Correlation (r)	p-value
SHBG vs. total testosterone	0.14	0.61
Age vs. total testosterone	-0.13	0.65

## Discussion

Main finding

Our study demonstrates the potential role of testosterone deficiency in younger males sustaining hip fractures. Among patients in our cohort who had testosterone levels analyzed on admission, 65% were found to have low levels despite most having no prior diagnosis of endocrine abnormalities or relevant clinical symptoms. These findings raise important clinical considerations regarding the evaluation and management of male patients presenting with fragility fractures.

The prevalence of comorbidities and lifestyle-related risk factors within our cohort is also noteworthy. Sixty-eight percent (n=42) of patients had multiple comorbidities, and 24 were on polypharmacy. Furthermore, a substantial proportion were active smokers (n=25; 41%), reported excessive alcohol consumption (n=17; 28%), or had a history of substance misuse (n=13; 21%). These factors are well-documented to be associated with reduced bone mineral density and increased fracture risk and may be related to the injuries observed in this cohort [12,18].

Role of testosterone

However, the presence of low testosterone in the majority of those tested suggests that hormonal deficiency may be an under-recognized yet significant contributor to bone fragility in this population. Testosterone is a key regulator of bone metabolism, promoting osteoblast activity and reducing bone resorption via both direct androgenic effects and aromatization to estradiol [19].

Hypogonadism is a well-established cause of secondary osteoporosis in older men and is associated with an increased risk of fragility fractures [20,21]. Nevertheless, this relationship remains underexplored in younger male populations. Given that only one of the 15 patients with low testosterone had a documented endocrine disorder, our findings suggest that undiagnosed hypogonadism may be more prevalent than currently appreciated in this age group presenting with a fragility hip fracture. Causes of low testosterone in this context can include both primary testicular failure and secondary hypogonadism from pituitary or hypothalamic dysfunction, obesity, chronic systemic illness, or medications such as opioids and glucocorticoids.

Given the high prevalence of smoking and excessive alcohol use in our cohort, it is notable that these lifestyle factors are also implicated in lowering testosterone levels. Chronic excessive alcohol intake can impair Leydig cell function and suppress the hypothalamic-pituitary-gonadal axis. At the same time, alcohol-related liver dysfunction may elevate sex hormone-binding globulin, thereby reducing bioavailable testosterone [22-24]. Such hormonal changes, combined with alcohol’s direct adverse effects on bone metabolism, may further compound fracture risk in this population.

These results have several clinical implications. At present, routine endocrine screening is not standard practice in younger men presenting with hip fractures. However, our results indicate that testosterone deficiency may be relatively common in this group. Identifying and managing hypogonadism could play a significant role in secondary fracture prevention. Potential interventions include lifestyle modification (e.g., smoking cessation and reduction of alcohol intake), ensuring adequate calcium and vitamin D intake, performing dual-energy X-ray absorptiometry (DEXA) scans to assess bone density, and initiating testosterone replacement therapy in appropriately selected patients.

Study limitations

This study has several limitations. The sample size was small, reflecting the rarity of hip fractures in men under 60. Furthermore, only 37.7% of the cohort had testosterone levels measured, introducing potential selection bias; those tested may have had additional clinical features prompting hormonal evaluation. Following the presentation of our findings, our local department has implemented new guidelines recommending routine testosterone screening in this patient population, with referral to endocrinology for further evaluation and management if levels are found to be low.

Other established risk factors for osteoporosis, such as smoking, alcohol use, corticosteroid exposure, and polypharmacy, were also prevalent in this group and may have independently contributed to fracture risk. Moreover, our study did not assess other important determinants of bone health, including serum vitamin D levels, bone turnover markers, dietary intake, or physical activity. Serial testosterone measurements were unavailable for most patients, limiting our ability to determine whether hypogonadism was transient (e.g., stress-induced) or persistent. However, as noted by Cappola et al. [25], although testosterone levels may decline acutely following a fracture, they often remain low relative to age-matched norms even during recovery, highlighting the need for longitudinal hormonal monitoring.

Future directions

Future research involving larger sample sizes and prospective study designs is needed to clarify the association between testosterone deficiency and fragility fractures in younger men. Comprehensive endocrine and metabolic evaluations, including serial testosterone measurements, DEXA scans, and assessments of lifestyle and nutritional factors, should be incorporated into standard fracture care protocols. Increased awareness among clinicians and closer multidisciplinary collaboration with endocrinology services may lead to earlier diagnosis of hypogonadism and facilitate interventions that could prevent subsequent fractures and improve long-term outcomes.

## Conclusions

Our findings suggest that low testosterone levels may be an under-recognized contributor to hip fractures in men under the age of 60, particularly among those with multiple comorbidities and lifestyle-related risk factors. Given the potential impact of hypogonadism on bone health, these results highlight the potential value of hormonal evaluation in this population. Failure to consider hormonal assessment may result in missed opportunities to identify a reversible risk factor for osteoporosis and to implement interventions that could reduce future fracture risk.

## References

[1] Johnell O, Kanis JA (2006). An estimate of the worldwide prevalence and disability associated with osteoporotic fractures. Osteoporos Int.

[2] (2025). Osteoporosis: assessing the risk of fragility fracture. https://www.nice.org.uk/guidance/cg146.

[3] Kanis JA, Cooper C, Rizzoli R, Reginster JY (2019). European guidance for the diagnosis and management of osteoporosis in postmenopausal women. Osteoporos Int.

[4] Watts NB, Adler RA, Bilezikian JP, Drake MT, Eastell R, Orwoll ES, Finkelstein JS (2012). Osteoporosis in men: an Endocrine Society clinical practice guideline. J Clin Endocrinol Metab.

[5] Kannegaard PN, van der Mark S, Eiken P, Abrahamsen B (2010). Excess mortality in men compared with women following a hip fracture. National analysis of comedications, comorbidity and survival. Age Ageing.

[6] Lin JC, Wu CC, Lo C (2014). Mortality and complications of hip fracture in young adults: a nationwide population-based cohort study. BMC Musculoskelet Disord.

[7] Vescini F, Chiodini I, Falchetti A (2021). Management of osteoporosis in men: a narrative review. Int J Mol Sci.

[8] Yeap BB, Alfonso H, Chubb SA (2020). U-shaped association of plasma testosterone, and no association of plasma estradiol, with incidence of fractures in men. J Clin Endocrinol Metab.

[9] Torremadé-Barreda J, Rodríguez-Tolrà J, Román-Romera I, Padró-Miquel A, Rius-Moreno J, Franco-Miranda E (2013). Testosterone-deficiency as a risk factor for hip fracture in elderly men. Actas Urol Esp (Engl Ed).

[10] Gaffney CD, Pagano MJ, Kuker AP, Stember DS, Stahl PJ (2015). Osteoporosis and low bone mineral density in men with testosterone deficiency syndrome. Sex Med Rev.

[11] Leifke E, Wichers C, Gorenoi V, Lucke P, von zur Mühlen A, Brabant G (2005). Low serum levels of testosterone in men with minimal traumatic hip fractures. Exp Clin Endocrinol Diabetes.

[12] MacLean C, Newberry S, Maglione M (2008). Systematic review: comparative effectiveness of treatments to prevent fractures in men and women with low bone density or osteoporosis. Ann Intern Med.

[13] Morales A, Bebb RA, Manjoo P (2015). Diagnosis and management of testosterone deficiency syndrome in men: clinical practice guideline. CMAJ.

[14] Emmerson BR, Varacallo MA, Inman D (2023). Hip fracture overview. StatPearls [Internet].

[15] Vermeulen A, Verdonck L, Kaufman JM (1999). A critical evaluation of simple methods for the estimation of free testosterone in serum. J Clin Endocrinol Metab.

[16] (2025). Free and bioavailable testosterone calculator. https://www.issam.ch/freetesto.htm.

[17] (2025). Is my study research?. https://www.hra-decisiontools.org.uk/research/.

[18] Abelsson A, Rystedt I, Suserud B-O, Lindwall L (2018). Learning high-energy trauma care through simulation. Clin Simul Nurs.

[19] Vanderschueren D, Laurent MR, Claessens F (2014). Sex steroid actions in male bone. Endocr Rev.

[20] Snyder PJ, Bhasin S, Cunningham GR (2016). Effects of testosterone treatment in older men. N Engl J Med.

[21] Wu FC, Tajar A, Pye SR (2008). Hypothalamic-pituitary-testicular axis disruptions in older men are differentially linked to age and modifiable risk factors: the European Male Aging Study. J Clin Endocrinol Metab.

[22] Emanuele MA, Emanuele N (2001). Alcohol and the male reproductive system. Alcohol Res Health.

[23] Rachdaoui N, Sarkar DK (2017). Pathophysiology of the effects of alcohol abuse on the endocrine system. Alcohol Res.

[24] Van Thiel DH, Gavaler JS, Lester R, Loriaux DL, Braunstein GD (1975). Plasma estrone, prolactin, neurophysin, and sex steroid-binding globulin in chronic alcoholic men. Metabolism.

[25] Cappola AR, Abraham DS, Kroopnick JM (2025). Sex-specific associations of vitamin D and bone biomarkers with bone density and physical function during recovery from hip fracture: the Baltimore Hip Studies. Osteoporos Int.

